# Some Aspects of the Chemistry of Alkynylsilanes

**DOI:** 10.1055/s-0036-1591979

**Published:** 2018-05-18

**Authors:** Gerald L. Larson

**Affiliations:** Gelest Inc.11 East Steel Road, Morrisville, PA 18940USAjlarson@gelest.com

**Keywords:** alkynes, azides, cross-coupling, enynes, protecting groups, silicon, Stille reaction

## Abstract

In amongst the considerable chemistry of acetylenes there lies some unique chemistry of alkynylsilanes (silylacetylenes) some of which is reviewed herein. This unique character is exemplified not only in the silyl protection of the terminal C–H of acetylenes, but also in the ability of the silyl group to be converted into other functionalities after reaction of the alkynylsilane and to its ability to dictate and improve the regioselectivity of reactions at the triple bond. This, when combined with the possible subsequent transformations of the silyl group, makes their chemistry highly versatile and useful.

1 Introduction

2 Safety

3 Synthesis

4 Protiodesilylation

5 Sonogashira Reactions

6 Cross-Coupling with the C–Si Bond

7 Stille Cross-Coupling

8 Reactions at the Terminal Carbon

9 Cross-Coupling with Silylethynylmagnesium Bromides

10 Reactions of Haloethynylsilanes

11 Cycloaddition Reactions

11.1 Formation of Aromatic Rings

11.2 Diels–Alder Cyclizations

11.3 Formation of Heterocycles

11.4 Formation of 1,2,3-Triazines

11.5 [2+3] Cycloadditions

11.6 Other Cycloadditions

12 Additions to the C≡C Bond

13 Reactions at the C–Si Bond

14 Miscellaneous Reactions

## 1 Introduction

Alkynylsilanes (silylacetylenes) as referred to in this review are those wherein the silyl moiety is directly bonded to the sp-carbon of the C≡C bond. Alkynylsilanes such as propargylsilanes are, therefore, not included. Acetylene chemistry has been extensively reviewed over the years. Several of the more recent additions are noted here. 1

**Gerald (Jerry) Larson:**
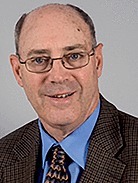
led Vice-President of R&D for Gelest Inc. for nearly 20 years before his retirement where he retains the position of Senior Research Fellow and Corporate Consultant. He received his B.Sc. degree in chemistry from Pacific Lutheran University in 1964 and his Ph.D. in chemistry (organic/inorganic) from the University of California-Davis in 1968. He served an NIH-postdoctoral year with Donald Matteson at Washington State University and a postdoctoral year with Dietmar Seyferth at MIT, after which he joined the faculty of the University of Puerto Rico-Río Piedras as an assistant professor in 1970 reaching full professor in 1979. He has been a visiting professor at various universities including Oregon State, Louisiana State, Universitá di Bari, Universität Würzburg, and Instituto Politécnico de Investigaciones de Mexico. On the industrial side, he rose to Vice-President of Research for Sivento, a Hüls group company, an antecedent of Evonik, after serving as Director of Applications, in Troisdorf, Germany. He is the author of over 130 publications and 30 patents. His hobbies include tennis, traveling and reading. He was born in 1942, as the first of three sons and a daughter, in Tacoma, Washington where he was raised on a small farm.

A significant portion of the applications of silylacetylenes occurs where the silyl group, typically trimethylsilyl, serves as a group for the protection of the reactive terminal C≡C–H bond. Supporting this silyl-protection strategy is that both the introduction and removal of the silyl group can be accomplished in high yield under a variety of mild conditions. The desilylation protocols are, in general, highly tolerant of other functional groups with the notable exception of silyl-protected alcohols. The reader will note several examples in this review where the silyl group basically provides a protective function, but has further synthetic potential. A further advantage of the terminal silylacetylenes is that the presence of the silyl group, for both steric and electronic reasons, can often influence the regio- and stereochemistry­ of reactions at the C≡C bond. This is most often reflected in cyclization reactions and it bears remembering that the regioselectively placed silyl group has the potential to be another group including hydrogen. Finally, the trimethylsilyl group has its own reactivity in the final product of a reaction at the C≡C bond. These often result in the generation of a vinylsilane unit, which can be further reacted under a number of conditions including protiodesilylation to the parent alkene. 2 Examples of these aspects of the chemistry are to be found throughout the review.

## 2 Safety

A report of an explosion using (trimethylsilyl)acetylene in an oxidative coupling under Glaser–Hay conditions was published. 3 After a thorough investigation the cause of the explosion was attributed to static electricity between the syringe needle used to introduce the copper catalyst and a digital thermometer inside the flask and not the thermal instability of the silane. It is interesting to note that the trimethylsilyl group can impart stability to alkynyl systems. A good example of this is 1,4-bis(trimethylsilyl)buta-1,3-diyne, which shows excellent thermal stability compared to that of the parent buta-1,3-diyne.

## 3 Synthesis

A well-known and often used approach to silylacetylenes is via the straightforward acid-base metalation, typically with RMgX or *n* -BuLi (the base), of a terminal acetylene (the acid) followed by reaction with an appropriate chlorosilane or related reactive organosilane. As a specific example, 1-(triisopropylsilyl)prop-1-yne was prepared by lithiation of propyne followed by reaction with triisopropylsilyl triflate (Scheme 1 ). 4


**Scheme 1** Example of a typical synthesis of a silylacetylene


The direct trimethylsilylation of a terminal alkyne can be carried out in a single step with the combination of LDA and TMSCl at low temperature. This was applied to the synthesis of **1** , which was used in a synthesis of complanadine A (Scheme 2 ). 5


**Scheme 2** Preparation of a silylacetylene employed in a synthesis of complanadine A


Marciniec and co-workers have demonstrated the direct silylation of terminal acetylenes using an iridium carbonyl catalyst and iodotrimethylsilane in the presence of Hünig’s base. 6 The yields are excellent and the process works well for diynes and is tolerant of OH and NH _2_ groups, albeit these end up as their trimethylsilylated derivatives in the final­ product (Scheme 3 ).


**Scheme 3** Ir-catalyzed direct trimethylsilylation of terminal alkynes


A direct dehydrogenative cross-coupling of a terminal alkyne and a hydrosilane provided a convenient and simple route to silylacetylenes. Thus, reaction of a terminal acetylene and a silane with a catalytic amount of NaOH or KOH gave the desired silylacetylene in high yield with expulsion of hydrogen. The reaction of a variety of acetylenes with dimethyl(phenyl)silane showed excellent general reactivity for 25 examples (Scheme 4 ). 7


**Scheme 4** Base-catalyzed direct dehydrogenative silylation of a terminal­ alkyne


## 4 Protiodesilylation

Because trialkylsilyl groups are very commonly used to protect the terminal C–H of an acetylene, protiodesilylation back to the parent acetylene is an important transformation. This can be accomplished under a number of mild reaction conditions. Among these is the simple reaction of (trimethylsilyl)acetylene derivatives with K _2_ CO _3_ /MeOH or, for more hindered silanes, TBAF/THF. Examples of these are to be found throughout this review. The selective protiodesilylation of (trimethylsilyl)acetylene group in the presence of an (triisopropylsilyl)acetylene group with K _2_ CO _3_ / THF/MeOH illustrates the potential for selective protection/deprotection (Scheme 5 ). 8


**Scheme 5** Selective deprotection of a (trimethylsilyl)acetylene group


1,4-Bis(trimethylsilyl)buta-1,3-diyne was metalated with one equivalent of MeLi and reacted with acrolein and subsequently protiodesilylated to yield vinyl diynyl carbinol **2** . Transmetalation of 1,4-bis(trimethylsilyl)buta-1,3-diyne with five equivalents of MeLi and reaction with acrolein gave the diol **3** in excellent yield. 9 These key intermediates were carried forth in syntheses of (+)- and (–)-falcarinol and (+)- and (–)-3-acetoxyfalcarinol (Scheme 6 ). 9


**Scheme 6** Selective metalation and protiodesilylation of 1,4-bis(trimethylsilyl)buta-1,3-diyne


## 5 Sonogashira Reactions

Of the many reactions at the terminal C–H of simple silylacetylenes, the Sonogashira reaction stands among the most important, where it has proved to be a very important synthetic entry into arylacetylenes and conjugated enynes. 10 These approaches typically make use of the Pd-catalyzed protocols employed in most cross-coupling reactions. The Au-catalyzed use of silylacetylenes in Sonogashira cross-coupling reactions has been reviewed. 11

Under the standard Sonogashira reaction conditions the C–Si bond does not react thus providing excellent protection of this position along with adding more desirable physical properties. Moreover, it provides an excellent entry into a variety of substituted silylacetylenes. Though the silyl group nicely provides protection of a terminal position in the Sonogashira cross-coupling, under modified conditions wherein the silyl group is activated, a Sonogashira-type conversion at the C–Si bond is possible, thus providing an alternative to a two-step protiodesilylation/Sonogashira sequence.


**Scheme 7** Sonogashira cross-coupling sequence employing desilylation


In an example of the use of the TMS group as a protecting group eventually leading to an unsymmetrically arylated system, 1-(trimethylsilyl)buta-1,3-diyne, prepared from 1,4-bis(trimethylsilyl)buta-1,3-diyne, was coupled with aryl iodide **4** to give the diyne **5** , which was protiodesilylated and further cross-coupled to give **6** , a potential hepatitis C NS5A inhibitor (Scheme 7 ). 12

Modest yields of symmetrical 1,4-diarylbuta-1,3-diynes resulted from the Sonogashira reaction of an aryl bromide and (trimethylsilyl)acetylene followed by treatment with NaOH/MeCN. The reaction sequence was the combination of the Sonogashira cross-coupling and a Glaser coupling in a two-step, single-flask operation. The second step did not require the further addition of catalyst. The reaction was tolerant of HO, CO _2_ H, and CHO functional groups (Scheme 8 ). 13


**Scheme 8** Sonogashira arylation and homocoupling without prior desilylation


The Beller group developed a copper-free protocol for the Sonogashira reaction with the more available and less costly aryl chlorides. Both (trimethylsilyl)acetylene and (triethylsilyl)acetylene reacted without loss of the silyl group. The key to the success of the reaction proved to be the sterically hindered ligand **7** (Scheme 9 ). 14


**Scheme 9** Copper-free Sonogashira cross-coupling


[3-Cyanopropyl(dimethyl)silyl]acetylene (CPDMSA, **8** ) was prepared and utilized in the synthesis of arene-spaced diacetylenes. The purpose of this particular silylacetylene was twofold, firstly it could be selectively deprotected in the presence of the (triisopropylsilyl)acetylene group and, secondly, it provided polarity allowing for a facile chromatographic separation of the key intermediates in the syntheses of the diethynylarenes (Scheme 10 ). The arene groups were introduced via Sonogashira cross-coupling. 15


**Scheme 10** Sonogashira cross-coupling and selective protiodesilylation


In a good example of the use of (trimethylsilyl)acetylene as a precursor to 1,2,4,5-tetraethynylbenzene, 1,2,4,5-tetraiodobenzene was reacted with (trimethylsilyl)acetylene under Sonogashira conditions to give 1,2,4,5-tetrakis[(trimethylsilyl)ethynyl]benzene. The trimethylsilyl groups were then converted into bromides with NBS in greater than 90% over the two steps. 1,2,4,5-Tetrakis(bromo­ethynyl)benzene was subsequently reacted with cyclohexa-1,4-diene to give 2,3,6,7-tetrabromoanthracene (Scheme 11 ). 16


**Scheme 11** Formation of 1,2,4,5-tetrakis(bromoethynyl)benzene


In related chemistry the direct ethynylation of tautomerizable heterocyclics under Sonogashira conditions without the need for conversion of the heterocyclic into an aryl halide was reported. These worked well for both (trimethylsilyl)acetylene and (triethylsilyl)acetylene (Scheme 12 ). 17


**Scheme 12** Direct Sonogashira-type ethynylation of tautomerizable heterocycles


In an interesting and useful approach, (trimethylsilyl)acetylene was cross-coupled with aryl iodides, bromides, and triflates in the presence of an amidine base and water. If water was omitted until the second stage of the reaction, i.e. reaction at the C–Si terminus, the result was the synthesis of unsymmetrical diarylacetylenes (Scheme 13 ). 18


**Scheme 13** Symmetrical and unsymmetrical diarylation of (trimethylsilyl)acetylene


The Sonogashira reaction of (trimethylsilyl)acetylene with 2,6-dibromo-3,7-bis(triflyloxy)anthracene was investigated as an intermediate in a route to anthra[2,3- *b* :6,7- *b* ′]difuran ( *anti* -ADT). In this reaction the Sonogashira cross-coupling occurred selectively at the triflate leaving the bromine groups available. This route did not, however, result in a synthetic approach to the desired anthracene difuran. Success was realized via the Sonogashira cross-coupling of (trimethylsilyl)acetylene with 2,6-diacetoxy-3,7-dibromoanthracene followed by desilylative cyclization. The thiofuran analogue, *anti* -ADT, was prepared via cross-coupling of **9** with (trimethylsilyl)acetylene, iodine cyclization, and reduction. A Suzuki–Miyaura cross-coupling and protiodesilylation gave the phenyl-substituted *anti* -ADT **10** . In an analogous manner the *anti* -diselenophene **12** was prepared from **11** in 62% yield over three steps (Scheme 14 ). 19

The relatively simple and economical catalyst system of FeCl _3_ / *N* , *N* ′-dimethylethylenediamine was used in the synthesis of 1-aryl-2-(triethylsilyl)acetylenes (6 examples, 40–90% yields). The reaction conditions were not mild, requiring 135 °C and 72 hours for completion (Scheme 15 ). 20

The Sonogashira reaction of several terminal alkynes with 1-fluoro-2-nitrobenzene gave 1-(2-nitrophenyl)-2-(triethylsilyl)acetylene. The use of (triethylsilyl)acetylene gave a considerably higher yield than other terminal alkynes. The TES group was not reacted further in this study (Scheme 16 ). 21


**Scheme 14** Sonogashira cross-coupling in the synthesis of thiophenes and selenophenes



**Scheme 15** Representative Sonogashira cross-coupling with iodopyridine



**Scheme 16** Sonogashira cross-coupling with 1-fluoro-2-nitrobenzene


## 6 Cross-Coupling with the C–Si Bond

Hatanaka and Hiyama were the first to report the cross-coupling of (trimethylsilyl)acetylenes. 22 This they accomplished with cross-coupling with β-bromostyrene to form conjugated enynes with TASF promotion. It bears mentioning that under the same conditions (trimethylsilyl)ethenes were cross-coupled in high yield with aryl and vinyl iodides (Scheme 17 ).


**Scheme 17** Conjugated enynes from (trimethylsilyl)acetylenes


Tertiary 3-arylpropargyl alcohols reacted with bis(trimethylsilyl)acetylene under Rh catalysis to give the hydroxymethyl-enyne regio- and stereoselectively with loss of benzophenone and one equivalent of the starting aryl­ethynyl group as its TMS-substituted derivative. Under Pd catalysis this silylated enyne could be cross-coupled with an aryl iodide, which was converted into the alkylidene-dihydrofuran. The alkylidene-dihydrofurans thus prepared exhibited fluorescent properties (Scheme 18 ). 23


**Scheme 18** Silyl Sonogashira cross-coupling of propargyl alcohols


Seeking a practical entry into 1,4-skipped diynes as potential precursors to polyunsaturated fatty acids, the Syngenta group investigated the cross-coupling of 1-aryl- or 1-alkyl-2-(trimethylsilyl)acetylene derivatives with propargyl chlorides. Under the best conditions the reaction of a (trimethylsilyl)acetylene with a propargyl chloride gave the 1,4-skipped diyne under promotion with fluoride ion and CuI catalysis. The method avoids the need for protiodesilylation to the parent acetylene, a requirement in other copper-catalyzed coupling protocols. The reaction failed with nitrogen-containing groups on the silylacetylene. The reaction proceeded well with 1-phenyl-2-(tributylstannyl)acetylene (70%) and 4-phenyl-1-(trimethylgermyl)but-1-yne (90%) (Scheme 19 ). 24

Denmark and Tymonko demonstrated the cross-coupling of alkynyldimethylsilanols with aryl iodides under promotion with potassium trimethylsilanolate (Scheme 20 ). 25 This protocol avoids the typical necessity of fluoride ion promotion and the associated disadvantages of cost and low tolerance for silicon-based protecting groups. The alkynylsilanols were prepared in a two-step reaction sequence. Interestingly, a direct comparison of the reaction rates of hept-1-yne, hept-1-ynyldimethylsilanol, and 1-(trimethylsilyl)hept-1-yne under the potassium trimethylsilanolate promotion conditions showed the hept-1-ynyldimethylsilanol to be considerably faster than hept-1-yne and the 1-(trimethylsilyl)hept-1-yne to be unreactive. This strongly suggests a role of the silanol group in the cross-coupling. A similar experiment with TBAF promotion showed all three to react with the silanol derivative being the fastest. Under the same conditions 4-bromotoluene gave a 25% conversion showing the advantages of using iodoarenes. 25 The TBAF-promoted cross-coupling of alkynylsilanols with aryl iodides had previously been shown. 26


**Scheme 19** Cross-coupling approach to 1,4-skipped diynes



**Scheme 20** Formation of ethynylsilanols and their cross-coupling with aryl iodides


The bis(trimethylsilyl)enyne **13** was nicely prepared via a Suzuki cross-coupling with 1-bromo-2-(trimethylsilyl)acetylene. The bis(trimethylsilyl)enyne **13** cross-coupled with aryl iodides in a sila-Sonogashira reaction to provide the silylated conjugated enyne **14** . Similar cross-coupling reactions of bis(trimethylsilyl)enyne **13** with vinyl iodides led to 1,5-dien-3-ynes **15** . Cyclic vinyl triflates also reacted well with bis(trimethylsilyl)enyne **13** to form 1,5-dien-3-ynes **16** (Scheme 21 ). 27


**Scheme 21** Suzuki cross-coupling with 1-bromo-2-(trimethylsilyl)acetylene and cross-coupling of the 1-(trimethylsilyl)alk-1-yne


## 7 Stille Cross-Coupling

(Trimethylsilyl)acetylene was deprotonated and reacted with tributyltin chloride to give 1-(tributylstannyl)-2-(trimethylsilyl)acetylene ( **17** ) in good yield (Scheme 22 ). 28


**Scheme 22** Synthesis of 1-(tributylstannyl)-2-(trimethylsilyl)acetylene


1-(Tributylstannyl)-2-(trimethylsilyl)acetylene ( **17** ) was prepared directly from (trimethylsilyl)acetylene and tributyltin methoxide in 49% isolated yield (Scheme 23 ). 29


**Scheme 23** Alternative synthesis of 1-(tributylstannyl)-2-(trimethylsilyl)acetylene


The bis(silyl)enyne **19** was prepared by cross-coupling 1-(tributylstannyl)-2-(trimethylsilyl)acetylene ( **17** ) with vinyl iodide **18** in 75% yield. In another approach to this end in the same paper, vinylstannane **20** reacted with 1-bromo-2-(trimethylsilyl)acetylene and 1-bromo-2-(triisopropylsilyl)acetylene to give the bis-silylated conjugated enynes **21** in good yield (Scheme 24 ). 30


**Scheme 24** Stille cross-coupling reactions


The alkynylation of the anomeric position of the benzyl-protected glucose derivatives **22** was accomplished with 1-(tributylstannyl)-2-(trimethylsilyl)acetylene ( **17** ) (Scheme 25 ). 31


**Scheme 25** sp ^3^ -sp Cross-coupling of 1-(tributylstannyl)-2-(trimethylsilyl)acetylene with a sugar derivative


1-(Tributylstannyl)-2-(trimethylsilyl)acetylene ( **17** ) was cross-coupled with **23** and found to be tolerant of a ketal and a cyclopropene. The TMS group was removed along with deacetoxylation of the ester upon treatment with K _2_ CO _3­_ /MeOH (Scheme 26 ). 32


**Scheme 26** Sonogashira cross-coupling showing functional group tolerance


1-(Tributylstannyl)-2-(trimethylsilyl)acetylene ( **17** ) was cross-coupled with the highly substituted aryl bromide **24** in a synthesis of (+)-kibdelone A. The TMS group was removed in 93% yield with AgNO _3_ ·pyridine in aqueous acetone (Scheme 27 ). 33


**Scheme 27** Stille cross-coupling of 1-(tributylstannyl)-2-(trimethylsilyl)acetylene with a highly substituted aryl bromide


Similarly to the Sonogashira reaction of (trimethylsilyl)acetylene, where the cross-coupling occurs at the C–H bond, the cross-coupling of 1-(tributylstannyl)-2-(trimethylsilyl)acetylene ( **17** ) occurs at the C–Sn bond rather than the C–Si bond. This was employed in the synthesis of the indole piece of sespendole (Scheme 28 ). 34


**Scheme 28** Stille cross-coupling of 1-(tributylstannyl)-2-(trimethylsilyl)acetylene with a highly substituted aryl triflate


In an approach to the synthesis of lactonamycins, a model glycine was prepared wherein a critical step was the addition of an ethynyl group onto a highly substituted arene. Thus, bromoarene **25** was subjected to a Stille cross-coupling with 1-(tributylstannyl)-2-(trimethylsilyl)acetylene ( **17** ) to give the ethynylarene **26** in 91% yield. This compared favorably with a three-step sequence (Scheme 29 ). 35


**Scheme 29** Selective Stille cross-coupling


## 8 Reactions at the Terminal Carbon

Under a co-catalysis approach, (triisopropylsilyl)acetylene reacted with enones to form β-ethynyl ketones in high yields (Scheme 30 ). The reaction worked well with ( *tert* -butyldimethylsilyl)acetylene and ( *tert* -butyldiphenylsilyl)acetylene as well, although (triethylsilyl)acetylene gave only 40% yield. Under the same reaction conditions the non-silylated terminal acetylenes phenylacetylene and oct-1-yne gave alkyne oligomerization. An asymmetric version of the reaction, which gave good yields (5 examples, 53–93%) and acceptable ee (81–90%), was also presented. 36


**Scheme 30** β-Ethynylation of α,β-unsaturated ketones


Carreira and co-workers reacted terminal acetylenes including (trimethylsilyl)acetylene with aldehydes in the presence of (+)- *N* -methylephedrine to give the propargyl alcohol in high yield and high ee (Scheme 31 ). 37


**Scheme 31** Asymmetric ethynylation of an aldehyde


The aldehyde **27** was reacted with (trimethylsilyl)acetylene under Carreira conditions to give a single diastereomer of **28** , which was O-silylated followed by protiodesilylation of the TMS group. This material was carried forth in a synthesis of hyptolide and 6- *epi* -hyptolide (Scheme 32 ). 38


**Scheme 32** Diastereoselective ethynylation of an aldehyde in a synthesis of hyptolide


In keeping with the common use of silylacetylenes as surrogates for the simple ethynyl organometallics, an ‘in situ’ process for the ethynylation of aldehydes was developed. In this chemistry a combination of ZnBr _2_ , TMSOTf, and Hünig’s base was used to generate the ethynylzinc reagent in situ and, along with a silylating agent, it was reacted with the aldehyde to generate the doubly silylated propargyl alcohol, which was O-deprotected with dilute hydrochloric acid (Scheme 33 ). 39


**Scheme 33** ‘In situ’ ethynylation of aldehydes


The aminomethylation of terminal alkynes was applied to a variety of acetylene derivatives including a single example with (triethylsilyl)acetylene, which provided the triethylsilylated propargyl amine in good yield. This was subsequently protiodesilylated and the resulting propargyl amine converted into a mixed bis(aminomethyl)alkyne in a 49% yield over three steps (Scheme 34 ). 40


**Scheme 34** Aminomethylation of terminal alkynes



**Scheme 35** Three-component reaction of norbornene with and (triisopropylsilyl)acetylene and an alkyne


(Triisopropylsilyl)acetylene was employed in a Ni-catalyzed, three-component reaction of the ethynylsilane, an alkyne, and norbornene. A variety of norbornene derivatives were reacted with good success. When (triisopropylsilyl)acetylene was used as the sole acetylene reactant, the bis(triisopropylsilyl)-1,5-enyne was produced. One example with a bicyclo[2.2.2]octene gave the corresponding product in only 12% yield when reacted with (triisopropylsilyl)acetylene (Scheme 35 ). 41

(Trimethylsilyl)acetylene could be directly alkylated to give 1-(trimethylsilyl)dodec-1-yne in modest yield. The yield of this sole silicon example was comparable to the direct alkylation of other terminal alkynes (Scheme 36 ). 42


**Scheme 36** Copper-catalyzed alkylation of (trimethylsilyl)acetylene


## 9 Cross-Coupling with Silylethynylmagnesium Bromides


**Scheme 37** Cross-coupling of silylethynylmagnesium bromide with anisoles


In a useful synthetic approach to alkynylsilanes (triisopropylsilyl)ethynylmagnesium bromide was cross-coupled with anisoles (23 examples 42–94% yield). In the cross-coupling of either 4-fluoroanisole or 4-cyanoanisole, the coupling of the F or CN substituent was favored over that of the methoxy group. The trimethylsilyl enol ether of cyclohexanone cross-coupled, as did 4,5-dihydrofuran. In one example the TIPS group was removed with TBAF/H _2_ O and the resulting acetylene cross-coupled in a Sonogashira reaction to the diarylacetylene (Scheme 37 ). 43

The bromomagnesium reagents of (triisopropylsilyl)acetylene ( **32** ) and ( *tert* -dimethylsilyl)acetylene were cross-coupled with primary and secondary alkyl iodides and bromides in a Sonogashira-type reaction employing the iron complex **33** . The reaction was tolerant of ester, amide, and aryl bromide groups (6 examples, 69–92% yield, 2 examples with TBS, both 83% yield). The free radical nature of the reaction was shown by the cross-coupling/cyclization of **34** (Scheme 38 ). 44

The synthesis of 2-alkylated ethynylsilanes was accomplished via a FeBr _2_ -catalyzed coupling reaction between a silylethynylmagnesium bromide reagent and a primary or secondary alkyl halide. This nicely broadens the scope of entries into 2-alkylated ethynylsilanes (Scheme 38 ). 45


**Scheme 38** sp ^3^ -sp Cross-coupling with silylethynylmagnesium bromide


## 10 Reactions of Haloethynylsilanes

A combination of the synthesis of TMS-, TIPS-, and CPDMS-substituted acetylenes and their cross-coupling with vinyl bromides and selective deprotection was effectively employed in the syntheses of callyberyne A ( **38** ) and callyberyne B ( **39** ). Thus, 1-iodo-2-(triisopropylsilyl)acetylene was converted into the skipped tetrayne **35** , (trimethylsilyl)acetylene was converted into enediyne **36** , and [(3-cyanopropyl)dimethylsilyl]acetylene was converted into dienyne **37** (Scheme 39 ). 8


**Scheme 39** Ethynylsilanes in the syntheses of callyberynes A and B


The Pd-catalyzed phenylation of 1-iodo-2-(trimethylsilyl)acetylene in a Kumada-type coupling reaction illustrated the potential of this route to 1-aryl-2-silylacetylenes. Numerous non-silicon terminated iodoalkynes were similarly arylated (Scheme 40 ). 46


**Scheme 40** Arylation of 1-iodo-2-(trimethylsilyl)acetylene


1-Iodo-3-(trimethylsilyl)acetylene was converted into (trimethylsilyl)ynamide **40** , which was subsequently protiodesilylated and the parent ynamide then converted into the iodoethynamide. In a more practical approach, (trimethylsilyl)acetylene and (triisopropylsilyl)acetylene were reacted in a two-step, single-flask protocol with NBS and a secondary amine to prepare the corresponding silylated ynamide. 47 48 The silylated ynamides were subsequently reported to be excellent precursors to highly substituted indolines (Scheme 41 ). 49

Danheiser and Dunetz were able to prepare ynamides from bromo- and iodoacetylenes, including 1-bromo-2-(trimethylsilyl)acetylene and 1-bromo-2-(triisopropylsilyl)acetylene. This work complements other approaches to substituted acetylenes. The protocol was extended to include cyclic carbamates, ureas, and sulfonamides, but not with silyl-substituted acetylenes. The key to the success of the reaction was the pre-formation of the amidocopper intermediate. 50 The resulting functionalized silylacetylenes could be readily protiodesilylated to the parent alkyne (Scheme 41 ). 48


**Scheme 41** Ethynylation of carbamates


The zinc reagent from **41** was reacted with either 1-iodo-2-(trimethylsilyl)acetylene or better with 1-bromo-2-(trimethylsilyl)acetylene to form 2-amino-5-(trimethylsilyl)pent-4-ynoate **42** , which was subsequently protiodesilylated and the parent acetylene cross-coupled to the 4-position of **43** in a total synthesis of the COPD (chronic obstructive pulmonary disease) biomarker, (+)-desmosine ( **44** ) (Scheme 42 ). 51


**Scheme 42** Zinc-catalyzed sp ^3^ -sp cross-coupling of 1-halo-2-(trimethylsilyl)acetylenes


Under indium catalysis 1-iodo-2-(trimethylsilyl)acetylene was reacted onto the anomeric carbon of glycals to furnish the α-ethynyl-2,3-unsaturated- *C* -glycoside. Only a single example employing 1-iodo-2-(trimethylsilyl)acetylene was reported. The trimethylsilyl group was converted into the iodide in 90% yield; this was in turn used in the preparation of a *C* -disaccharide bridged by an ethynyl group (Scheme 43 ). 52


**Scheme 43** Ethynylation of glycals


The advantages of the selective chemistry of different silyl groups was applied to the synthesis of tris(biphenyl-4-yl)silyl (TBPS) terminated polyynes. Based on the findings that bulky groups on the termini of polyynes provide stability and calculations showing the TBPS group to have over twice the radius of the TIPS group, this group was investigated in the synthesis and stability of TBPS-terminated polyynes. The synthesis of the polyynes started with the reaction of lithium (trimethylsilyl)acetylide with tris(biphenyl-4-yl)chlorosilane. Selective protiodesilylation gave the TBPS-substituted acetylene, and NBS bromination gave 1-bromo-2-[tris(biphenyl-4-yl)silyl]acetylene. This bromo derivative was cross-coupled with (trimethylsilyl)acetylene to give the mixed silylbuta-1,3-diyne, which was subjected to selective protiodesilylation and homocoupling to give 1,8-bis[tris(biphenyl-4-yl)silyl]octa-1,3,5,7-tetrayne in 77% over two steps. Iterations of these reactions were used to prepare the triyne **45** and hexayne **46** (Scheme 44 ). 53


**Scheme 44** Synthesis of polyynes


## 11 Cycloaddition Reactions

Silylacetylenes, like many alkynes, undergo an extensive variety of cycloaddition reactions. In many cases based on electronic and steric factors the silyl group can impart useful regio- and stereoselectivities in addition to the ability to chemically transform the silyl group to other useful functionalities.

### 11.1 Formation of Aromatic Rings

The tricyclization of alkynes to aromatic rings has long been recognized, as has the use of silylacetylenes in this practice. Silyl-protected arylacetylenes reacted with 2-(phenylethynyl)benzaldehyde under acid catalysis to produce the 2-aryl-3-silylnaphthalene in good yield. The TMS-protected arylalkynes resulted in the formation of 2-arylnaphthalene with protiodesilylation taking place under the reaction conditions. However, the more hindered TES-, TBS-, and TIPS-protected derivatives gave the corresponding 3-silylnaphthalenes allowing for the ICl *ipso* iodination of the silyl group to provide the iodonaphthalene for further elaboration via cross-coupling chemistry. The chemistry was applied to the synthesis of several highly encumbered polyaromatic systems (Scheme 45 ). 54


**Scheme 45** Cyclization to aromatic rings from arylacetylenes


The Rh-catalyzed reaction of (trimethylsilyl)acetylenes with cyclobutenols gave 1,2,3,5-tetrasubstituted benzenes with the trimethylsilyl group regioselectively positioned in the 2-position. No conversions of the trimethylsilyl group were carried out in this work (Scheme 46 ). 55


**Scheme 46** Cyclobutenol to a TMS-substituted arene


Methyl 3-(trimethylsilyl)propynoate was successfully employed in the synthesis of 2 *H* -quinolizin-2-ones. In this approach the trimethylsilyl group conveniently served the purpose of protecting the acidic hydrogen of the parent terminal acetylene (Scheme 47 ). 56


**Scheme 47** Quinolizin-2-ones from methyl 3-(trimethylsilyl)propynoate


The cationic rhodium catalyst [Rh(cod) _2_ ]BF _4_ /BIPHEP brought about the cyclotrimerization of (trimethylsilyl)acetylene and unsymmetrical electron-deficient acetylenes. Unfortunately, neither the stoichiometry nor the regioselectivity of the cyclization was optimal. Larger silyl groups tended to favor the addition of one of the silylacetylene moieties and two of the electron-deficient alkynes, whereas increasing the steric bulk of the electron-deficient alkyne resulted in the reaction of two equivalents of the silylacetylene. (Triisopropylsilyl)acetylene failed to react. Protiodesilylation of a mixture of regioisomers was able to simplify the reaction mixture, but reaction with ICl gave a synthetically challenging mixture of isomers in modest yield (Scheme 48 ). 57


**Scheme 48** Mixed substituted arenes from cross-cyclization of (trimethylsilyl)- and (triethylsilyl)acetylene with ethyl but-2-ynoate


The cyclotrimerization of ethyl 3-(trimethylsilyl)propynoate gave **47** as a single regioisomer in 92% yield (Scheme 49 ). 58


**Scheme 49** Homocyclization of ethyl 3-(trimethylsilyl)propynoate


Complete regioselection in the formation of 2-aryl-1,3,5-tris(silyl)benzene was realized in the Pd-catalyzed reaction of two equivalents of a terminal alkyne, including (trimethylsilyl)acetylene, and an equivalent of a β-iodo-β-silylstyrene. The nature of the silylstyrene proved crucial as trialkylsilyl (TMS, TES, TBS, Me _2_ BnSi) groups gave poor yields and the phenylated silyl groups gave better yields, with the β-Ph _2_ MeSi-substituted styrene proving optimal. Selective electrophilic substitution of the 5-(trimethylsilyl) group, *para* relative to the aromatic substituent, proved possible. In a demonstration of the potential synthetic utility of the highly silylated systems, a number of conversions of the silyl groups were carried out including protiodesilylation, acylation, iodination, and Denmark cross-coupling. It is noteworthy that the iododesilylation of **48** was selective for the formation of **49** and that iododesilylation of a phenyl group from the Ph _2_ MeSi group did not occur. Comparable selectivity was noted in the acetylation of **48** to 4-phenylacetophenone (Scheme 50 ). 59


**Scheme 50** Cyclotrimerization with a vinyl iodide and subsequent conversions­


### 11.2 Diels–Alder Cyclizations

Silylacetylenes were shown to provide excellent regiochemical control in the cobalt-catalyzed Diels–Alder reaction with 1,3-dienes. In the unsubstituted case various (trialkylsilyl)- and (triphenylsilyl)acetylenes were reacted with 2-methylbuta-1,3-diene under cobalt catalysis. The regioselectivity was highly dependent on the accompanying ligand employed with CoBr _2_ (py-imin) [py-imin = *N* -mesityl-1-(pyridin-2-yl)methanimine, **56** ] favoring the *meta* regioisomer **50** after DDQ oxidation to the aromatic derivative. On the other hand, the use of CoBr _2_ (dppe) [dppe = 1,2-bis(diphenylphosphino)ethane] favored the *para* isomer **51** . In addition a number of 1-(trimethylsilyl)alk-1-ynes were reacted with 2-methylbuta-1,3-diene. Here the yields were very high, but the regioselectivity was less than that observed with the simple silylacetylenes. Of particular interest was the result from the reaction of 3-(trimethylsilyl)propargyl acetate with Danishefsky’s diene, 2-(trimethylsiloxy)buta-1,3-diene (Scheme 51 ). 60


**Scheme 51** Diels–Alder cyclization of silylacetylenes with 1,3-dienes


The synthesis of aryl and vinyl iodides has taken on increased importance due to their facility as electrophilic partners in various cross-coupling reactions. Building on the Diels–Alder chemistry of butadienes with (trimethylsilyl)acetylenes, the Hilt group devised an efficient route to highly substituted aryl iodides wherein the TMS group served nicely to define the regiochemistry and provide the iodide functionality. The complete reaction sequence could be carried out in a single flask although considerable effort was placed on the oxidation/iodination step. For example, ICl/CH _2_ Cl _2_ gave only 5% of the iodide **54** , NIS/MeCN gave modest yields of the iodide in 5 cases, but the reaction was very slow and product decomposition led to purification difficulties. The combination of H _2_ O _2_ /ZnI _2_ gave modest yields, but again in a slow reaction that required further oxidation with DDQ for completion. Finally, the use of *tert* -butyl­ hydroperoxide with ZnI _2_ and K _2_ CO _3_ was found to give high yields of the desired iodides (Scheme 52 ). 61


**Scheme 52** Diels–Alder cyclization to cyclic 1,4-dienes


### 11.3 Formation of Heterocycles

The diynes **57** were subjected to cyclotrimerization with hex-1-yne; the TMS-substituted derivative (R = TMS) gave considerably better yields and regioselectivities than the protonated analogues (R = H). Interestingly, the application of this cyclotrimerization towards the synthesis cannabinols employed the use of 3-(trimethylsilyl)prop-1-yne (instead of hex-1-yne), which showed clean regioselectivity to give **61** from **60** . The bis(trimethylsilyl)arene **61** was protiodesilylated to **62** , which was carried through to cannabinol ( **63** ) (Scheme 53 ). 62


**Scheme 53** Cyclizations leading to cannabinols


Under strong base catalysis, 1-aryl-2-silylacetylenes were converted into oxasilacyclopentenes upon reaction with aldehydes or ketones. The reaction required that the silyl moiety contain a Si–H bond [SiHMe _2_ , SiH( *i* -Pr) _2_ , SiHPh _­2_ ]. Among the catalysts investigated KO *t* -Bu was clearly superior, with fluoride ion sources tending to give more of the product of direct alkynylation of the carbonyl. Silylalkynylation of the carbonyl followed by base-catalyzed intramolecular hydrosilylation of the C≡C bond is proposed. 4-Methoxyphenyl- and 2-tolyl-substituted (dimethylsilyl)acetylenes on reaction with cyclohexanone gave only alkynylation of the ketone, but 4-fluorophenyl- and 4-(trifluoromethyl)phenyl-substituted (dimethylsilyl)acetylenes gave good yields of their respective oxasilacyclopentenes (8 examples, 48–87% yields). The oxasilacyclopentene **64** was shown to have synthetic utility as it could be oxidized, epoxidized, and cross-coupled all in good yield (Scheme 54 ). 63


**Scheme 54** Oxasilacyclopentenes via cyclization with ketones


Cyclotrimerization of **65** (R = TMS) with 4-hydroxypentanenitrile gave the desired product regioselectivity, albeit in only 42% yield, this compared to 83% yield from the parent diyne **66** (R = H) (Scheme 55 ). 64


**Scheme 55** Cyclization of a silylated skipped diyne with a nitrile


Whereas the Ru-catalyzed reaction of an internal alkyne, carbon monoxide, and an enone produced hydroquinones in a [2+2+1+1]-cycloaddition reaction, (trimethylsilyl)acetylenes reacted in a [3+2+1] fashion to form an α-pyrone, wherein the carbonyl and α-carbon of the enone provided three atoms. The resulting 3-(trimethylsilyl)-2 *H* -pyran-2-ones were not elaborated further (Scheme 56 ). 65 66


**Scheme 56** Carbonylative cyclization with an enone



**Scheme 57** Cyclization to isoxazoles


The reaction of 1-(methoxydimethylsilyl)-2-phenyl­acetylene with propanenitrile oxide, generated in situ from 1-nitropropane and phenyl isocyanate, gave a mixture of 4- and 5-silylated isoxazoles favoring formation of the 4-silyl isomer. Acid hydrolysis of this mixture allowed isolation of the pure 4-dimethylsilanol derivative in 49% overall yield. In a similar manner the ‘in situ’ generated benzonitrile oxide reacted to give, after hydrolysis, the corresponding 4-silanol products. These silanols were subjected to Denmark cross-coupling protocols to take advantage of the position of the silyl group to introduce aryl substituents at the 4-position of the isoxazole. Unfortunately, in addition to the cross-coupling reaction product, a considerable amount of protiodesilylated isoxazole was also generated (Scheme 57 ). 67

In a study involving the addition of 2-substituted pyridines with 3-substituted propargyl alcohols to give indolizines, 1-phenyl-3-(trimethylsilyl)prop-2-yn-1-ol was reacted with ethyl 2-pyridylacetate to give the TMS-substituted indolizine **69** (Scheme 58 ). The TMS group was not reacted further in this work. 68

The acid-catalyzed reaction of 1-phenyl-3-(trimethyl­silyl)prop-2-yn-1-ol with a series of primary amides gave 2,4-disubstituted 5-[(trimethylsilyl)methyl]oxazoles in excellent yields. The preferred Brønsted acid for this useful conversion was PTSA (Scheme 58 ). 69


**Scheme 58** Cyclization with 3-(trimethylsilyl)propargyl alcohols


1-Alkyl- or 1-aryl-substituted 2-(trimethylsilyl)acetylenes were used as alternatives to terminal acetylenes in the synthesis of tetrahydropyridines (18 examples, 54–96% yield; dr 20:1). In this approach the presence of the trimethylsilyl group also facilitated the generation of an azomethine ylide, which could be further converted. Thus, reaction of an α,β-unsaturated imine with the 1-alkyl- or 1-aryl-substituted 2-(trimethylsilyl)acetylenes gave the dienyl imine, which underwent an intramolecular aza-cyclization reaction. The resulting 2-silyl-1,2-dihydropyridine was reductively desilylated to the tetrahydropyridine, reacted with an alkyne to give a tropane derivative (7 examples, 48–83% yield, dr 15:1 to 20:1, or reacted via a desilylative electrocyclization to give a 2-azabicyclo[3.1.0] system (Scheme 59 ). 70


**Scheme 59** Cyclization of 1-alkyl- or 1-aryl-substituted 2-(trimethylsilyl)acetylenes with α,β-unsaturated imines and subsequent reactions


The reaction of thioisotin with 1-(trimethylsilyl)prop-1-yne gave a single regioisomeric -3-(trimethylsilyl)-4 *H* -benzothiopyran-4-one in a decarbonylative cyclization process. The reaction with (trimethylsilyl)acetylene, however, provided a 6:1 mixture of regioisomers (Scheme 60 ). 71


**Scheme 60** Cyclization of 1-(trimethylsilyl)prop-1-yne with thioisotin


In an interesting cyclization *N* -(2-cyanophenyl)- *N* -phenylbenzamides were reacted with internal acetylenes to give quinolones. When 1-(trimethylsilyl)prop-1-yne was employed the trimethylsilyl group was placed on the 4-position with high regioselectivity as compared to that of the *tert* -butyl analogue (Scheme 61 ). 72


**Scheme 61** Decyanative cyclization of 1-(trimethylsilyl)prop-1-yne with *N* -(2-cyanophenyl)- *N* -phenylbenzamides


The Ni-catalyzed [4+2] cycloaddition of an internal alkyne with an azetidin-3-one resulted in the formation of various piperidines. Interestingly, the (trimethylsilyl)acetylene derivatives employed showed reversed regioselectivity to those of the *tert* -butyl and trimethylstannyl analogues. Although the carbonyl and Boc groups were reduced with LiAlH _4_ , reactions of the trimethylsilyl group were not attempted on these systems. When phenylacetylene derivatives were reacted, 1-phenyl-2-(trimethylsilyl)acetylene gave the same regioselectivity as 1-(trimethylsilyl)prop-1-yne, but 1-phenyl-2-(trimethylstannyl)acetylene and 1-(trimethylstannyl)prop-1-yne reversed their regioselectivity. A total of four different (trimethylsilyl)acetylene derivatives was investigated (Scheme 62 ). 73


**Scheme 62** Cyclization of (trimethylsilyl)acetylene derivatives with azetidinones


In an approach to complanadine A and various lycodine derivatives the Siegel group, 1,4-bis(trialkylsilyl)buta-1,3-diynes were used in a [2+2+2] cycloaddition strategy. Thus, the key intermediate cyanoalkyne **75** was prepared on a gram scale and reacted with three different 1,4-bis(trialkylsilyl)buta-1,3-diynes; 1,4-bis(trimethylsilyl)buta-1,3-diyne gave the best yield of the 2-alkynylated pyridine **76** when the reaction was carried out with CpCo(CO) _2_ as catalyst. A small amount of the (trimethylsilyl)ethynyl group was protiodesilylated upon silica gel chromatography and **76** was cleanly protiodesilylated upon treatment with TBAF/THF to **77** . Trimethylsilylation of the terminal alkyne **77** then provided alkynylsilane **78** , which was subjected to the CpCo­(CO) _2_ -catalyzed [2+2+2] cycloaddition with **75** . This provided the undesired 2,2′-bipyridine derivative in a modest 43% yield. After considerable study and effort it was found that modification of the cyanoalkyne **75** to the *N* -formyl-cyanoalkyne **79** and reaction with **78** with added triphenylphosphine and under very dilute 5 mM conditions gave an acceptable yield of the desired 2,3-bipyridyl structure **80** , which was protiodesilylated and deprotected to complanadine A (Scheme 63 ). In model studies several 1-aryl-2-(trimethylsilyl)acetylenes were reacted with **75** to give the 2-aryl-3-(trimethylsilyl) cycloaddition products in low to modest yields. In none of these cases was the trimethylsilyl group reacted further. A facile conversion of **75** into lycodine was presented wherein the cycloadditions was carried out with bis(trimethylsilyl)acetylene followed by protiodesilylation and deprotection in a 24% overall yield (Scheme 63 ). 5 74


**Scheme 63** Cyclizations of alkynylsilanes with alkyne functional nitriles


1,4-Bis(trimethylsilyl)buta-1,3-diyne is thermally stable and, therefore, serves as an excellent substitute for the thermally sensitive buta-1,3-diyne. It was employed in a [2+2+2] cyclization with the alkynyl nitrile **75** . The reaction was extended to 1-aryl-2-(trimethylsilyl)acetylenes, wherein the trimethylsilyl group dictated the regioselectivity to place the trimethylsilyl group on the 3-position of the pyridine ring formed. The yields were modest, ranging from <5% to 62% over 9 examples (Scheme 63 ). 5

### 11.4 Formation of 1,2,3-Triazines

A series of 1,4-disubstituted 1,2,3-triazines **84** was prepared in a one-pot, three-step sequence involving first a Sonogashira preparation of a 1-aryl-2-(trimethylsilyl)acetylene from (trimethylsilyl)acetylene, reaction with an alkyl azide and, finally, deprotection of the 5-trimethylsilyl group (Scheme 64 ). 75

1-Aryl-2-(trimethylsilyl)acetylenes, readily formed via a Sonogashira reaction from (trimethylsilyl)acetylene, reacted with sodium azide and an alkyl bromide in a three-step, one-pot sequence to yield a desilylated 1-alkyl-4-aryl-1,2,3-triazole **85** or **86** . The reaction took place via initial deprotection of the trimethylsilyl group followed by the [3+2] click cycloaddition. This represents a safe and scalable process for the formation of 1,4-disubstituted 1,2,3-triazoles (Scheme 64 ). 76

The reaction of 1-(trimethylsilyl)alk-1-ynes with CuBr/Et _3_ N served to directly prepare the alkynylcopper reagent without prior desilylation. The resulting copper reagent underwent reaction with various azides to form the 1,2,3-triazenes **87** in excellent yields. When the reaction was carried out with (trimethylsilyl)acetylene or (triisopropylsilyl)acetylene, the reaction occurred at the C–H terminus. TIPS- and TBS-terminated acetylenes failed to react (Scheme 64 ). 77

The dichloropyridazine **88** was converted into the [1,2,3]triazole-fused pyrazinopyridazinedione **89** in a three-step sequence with ethyl 3-(trimethylsilyl)propynoate. The TMS group was lost in the last step of the sequence, but provides the desired regioselectivity in the azide click step of the sequence (Scheme 64 ). 78


**Scheme 64** Formation of 1,2,3-triazoles via click chemistry on alkynylsilanes


### 11.5 [2+3] Cycloadditions

The reaction of ethyl and methyl 3-(trimethylsilyl)propynoate with 2-formylphenylboronic acid under [Rh(OH)(cod)] _2_ catalysis gave 3-(trimethylsilyl)-1 *H* -inden-1-ols **90** in high yield and with high regioselectivity. Similar results were realized with 2-acetylphenylboronic acid. 1,4-Bis(trimethylsilyl)buta-1,3-diyne reacted with 2-formylphenylboronic acid to give the enyne **92** in high yield (Scheme 65 ). 79

In a related approach 2-bromo- and 2-chlorophenylboronic acids underwent a carbonylative cycloaddition with various alkynes including (trimethylsilyl)acetylenes to give 1 *H* -inden-1-ones; the reaction was catalyzed by RhCl(cod) _2_ . With the exception of ethyl 3-(trimethylsilyl)propynoate, the regioselectivity was very high. 1 *H* -Inden-1-ones were also formed via the reaction of 2-bromophenylboronic acid, a (trimethylsilyl)acetylene, and paraformaldehyde, although the reaction took longer and required a higher temperature (Scheme 65 ). 80


**Scheme 65** [2+3] Cycloadditions of silylacetylenes with 2-functionalized phenylboronic acids


Benzoyltrimethylsilanes reacted with (trimethylsilyl)acetylenes under Au catalysis to form indan-1-ones. Mechanistic studies showed that a migration of the acylsilyl group to the C≡C bond occurred to form the 2-(trimethylsilyl)indan-1-one; the trimethylsilyl group was lost upon workup. On the other hand the more sterically hindered and stable benzoyl( *tert* -butyl)dimethylsilane gave the 2-( *tert* -butyldimethylsilyl)-substituted indanone. The reaction proceeds through the formation of the interesting 2-(trimethylsilyl)-substituted silyl enol ether (Scheme 66 ). 81


**Scheme 66** [2+3] Cycloadditions of silylacetylenes with benzoylsilanes


### 11.6 Other Cycloadditions

A three-component co-cyclization involving ethyl cyclopropylideneacetate, a 1,3-diyne, and a heteroatom-substituted acetylene gave highly functionalized cyclohepta-1,3-dienes. The 1,3-diynes reacted at only one of the C≡C bonds. When 1-(trimethylsilyl)deca-1,3-diyne was reacted, the hexyl-substituted C≡C bond was the one that reacted to give the cycloheptadiene ring. Protiodesilylation provided the terminal acetylene with concomitant formation of the enone moiety. A competition experiment using equimolar amounts of ethyl cyclopropylidene acetate, 1-ethynyl-2-(trimethylsilyl)-1 *H* -pyrrole, and 1,4-bis(trimethylsilyl)buta-1,3-diyne and hexadeca-7,9-diyne resulted in the reaction of the hexadecadiyne to the exclusion of the bis(trimethylsilyl)butadiyne (Scheme 67 ). 82


**Scheme 67** Mixed diyne cyclization with ethyl cyclopropylideneacetate



**Scheme 68** Formation of silylated fulvenes


A Rh-catalyzed [2+2+1] cross-cyclotrimerization of (triisopropylsilyl)acetylene with propynoate esters gave the silyl-substituted fulvene in modest to excellent yield. The use of (triethylsilyl)-, ( *tert* -butyldimethylsilyl)-, and ( *tert* -butyldiphenylsilyl)acetylenes gave poor yields of the cyclic trimer. *N* , *N* -Dimethylbut-2-ynamide and (triisopropylsilyl)acetylene gave a very poor yield of fulvene product, with ethynylation of the C≡C bond as the predominant pathway. The silylfulvene was reductively complexed with Rh(III) to give the rhodium dimer **93** (Scheme 68 ). 83

## 12 Additions to the C≡C Bond

The Ru-catalyzed hydroacylation of 4-methoxybenzaldehyde with 1-(trimethylsilyl)prop-1-yne gave a mixture of isomeric trimethylsilyl dienol ethers **94** and **95** . 84 The reaction of a tertiary amine with methyl 3-(trimethylsilyl)propynoate gave addition of the amine to the C≡C bond and the formation of an allenoate ion. This, in the presence of an arylaldehyde, gave predominantly bis-addition of the aldehyde resulting in two products **96** and **97** ; aliphatic aldehydes gave addition at the C–H terminus of the C≡C bond to give **98** . No reaction occurred with ethyl but-2-ynoate indicating that the trimethylsilyl group was essential (Scheme 69 ). 85


**Scheme 69** Aldehyde addition to an alkynylsilane


(Trimethylsilyl)acetylenes were reacted under Ni catalysis with phthalimides to give decarbonylation and alkylidenation of one of the carbonyl groups. Although the reaction appears to be potentially general, all but two of 11 examples were with *N* -(pyrrolidino)phthalimide. The use of a catalytic amount of the strong and sterically demanding methylaluminum bis-(2,6-di- *tert* -butyl-4-methylphenoxide) (MAD) was crucial in the success of the reaction. In the absence of MAD the major products were isoquinolones. Various 1-alkyl and 1-aryl-substituted (trimethylsilyl)acetylenes were utilized and gave the *E* -isomer as the product, but only 1-phenyl-2-(trimethylsilyl)acetylene and 1-(4-methoxyphenyl)-2-(trimethylsilyl)acetylene gave mixtures of *Z* - and *E* -isomers. Two additional examples of reactions where the silyl groups were PhMe _2_ Si and TBS were successful, albeit in lower yield. Two internal alkynes failed to react indicating that the presence of the TMS group is necessary for the reaction (Scheme 70 ). 86 87


**Scheme 70** Decarbonylative addition to a silylacetylene


The olefination of ynolates was accomplished with 3-silylpropynoates giving excellent selectivity for the *E* -enyne. Ag-catalyzed cyclization of the resulting enynes was carried out to give either the 5- *exo* -tetronic acid derivatives or the 6- *endo* -pyrones. The triethylsilyl-tetronic acid **99** was stereoselectively converted into the corresponding iodide **100** , which was in turn subjected to phenylation via a Suzuki cross-coupling and to ethynylation via Sonogashira cross-coupling (Scheme 71 ). 88


**Scheme 71** Addition to silylpropynoates and reaction of the resulting vinylsilanes


A series of silylated propargylic alcohols was prepared via the straightforward reaction of a lithiated silylacetylene and a variety of aromatic and aliphatic aldehydes and ketones. These silylated propargylic alcohols were then subjected to the Meyer–Schuster rearrangement to give acylsilanes; propargyl alcohols derived from aromatic aldehydes underwent the rearrangement in good yield under catalysis with either PTSA·H _2_ O/ *n* -Bu _4_ N·ReO _4_ or Ph _3_ SiOReO _3_ . The PTSA·H _2_ O/ *n* -Bu _4_ N·ReO _4_ system did not work for electron-donating aryl systems, though the Ph _3_ SiOReO _3_ catalyst worked well for these. Propargyl alcohols derived from aliphatic aldehydes failed to give acylsilanes with the exception of pivaldehyde. Propargylic alcohols derived from diaryl ketones gave either indanones or acylsilanes (Scheme 72 ). 89


**Scheme 72** Rearrangement and oxidation of silylpropargyl alcohols


A one-step hydroiodination of 1-aryl-2-silylacetylenes to the vinyl iodide, highly useful substrates for cross-coupling applications, was found to occur upon treatment of the 1-aryl-2-silylacetylenes with iodotrimethylsilane. The reaction sequence of a Sonogashira cross-coupling of (trimethylsilyl)acetylene and an aryl halide followed by the hydroiodination resulted in a facile synthesis of α-iodostyrene derivatives; the reaction resulted in the Markovnikov addition of HI to the C≡C bond. It was further found that the terminal acetylene itself would undergo the reaction as well. More hindered silyl groups gave a lower yield of the vinyl iodide (Scheme 73 ). 90


**Scheme 73** Hydroiodination of alkynylsilanes


A three-component with methyl 3-(trimethylsilyl)propynoate, an amine, and an imine is directed by both the ester and the trimethylsilyl moieties. The reaction involves a 1,4-silyl shift. When salicyl imines were used as substrates the products were chromenes. This reaction was shown to proceed through the aminal **101** , which could be trapped with allyltrimethylsilane or the TMS enol ether of aceto­phenone (Scheme 74 ). 91


**Scheme 74** Reaction of 3-silylpropynoates with imines


A variety of 3-silylpropynals and silylethynyl ketones, prepared via a silylation, deprotection, oxidation sequence, were converted into 2-silyl-1,3-dithianes, which are useful synthons via their potential for anion relay chemistry (ARC). 92 Although 8 different silyl groups showed good results, the dithiation did not occur when the silyl was sterically hindered, as with TBDPS, TIPS, *t* -Bu _2_ HSi, or *i* -Pr _2_ HSi (Scheme 75 ). 93


**Scheme 75** Dithiation of silylpropynals


The lithium aluminum hydride reduction of 4-silylbut-3-yn-2-ones provided the 4-silylbut-3-en-2-ol in good yields and high *E* / *Z* ratios (Scheme 76 ). 94


**Scheme 76** Lithium aluminum hydride reduction of silylpropargyl alcohols­


The β-silyl effect to stabilize β-cationic intermediates was employed in the regioselective addition of ICl to silylacetylenes. The diastereoselectivity of the addition is the opposite of that found for the reaction of ICl with the simple terminal alkyne. The *Z* / *E* selectivity is higher with aryl-substituted silylacetylenes, though the *Z* selectivity of alkyl-substituted silylacetylenes increases with an increase in the size of the silyl group (Scheme 77 ). 95


**Scheme 77** Iodochlorination of silylacetylenes


The addition of the halogens to (trimethylsilyl)acetylene in the absence of light produced the *E* isomer, which could be equilibrated to a mixture of both stereoisomers. In the cases of the *E* -dichloride or *E* -dibromide the equilibration was brought about by exposure to light in the presence of a trace of bromine. In the case of the *E* -diiodide, prolonged refluxing in cyclooctane produced an *E* / *Z* mixture of 9:1 (Scheme 78 ). 96


**Scheme 78** Halogenation of (trimethylsilyl)acetylene


The reaction of Weinreb amides with internal acetylenes promoted by a Kulinkovich-type titanium intermediate gave α,β-unsaturated ketones in modest yield. The reaction conditions were mild with activation of the titanium promoter as the last step at room temperature. With TMS-terminated acetylenes, the yields were comparable to those of other alkynes investigated, though with slightly lower regioselectivity­ (Scheme 79 ). 97


**Scheme 79** Reaction of Weinreb amide with silylacetylenes


The *syn* addition of two aryl groups from an arylboronic acid to an internal alkyne resulted in the formation of 1,2-disubstituted 1,2-diarylethenes. In the single example using a silylacetylene, the reaction of ethyl 3-(trimethylsilyl)propynoate with *p* -tolylboronic acid under Pd catalysis gave the highly substituted ethyl 2,3-di( *p* -tolyl)-3-(trimethylsilyl)propenoate via the addition of two equivalents of the *p* -tolyl group (Scheme 80 ). 98 99

The highly regio- and stereoselective addition of a boronic­ acid to silylacetylenes occurred under mild conditions and in high yields. Interesting points were that 1-(triethylsilyl)hex-1-yne was more regioselective than (trimethylsilyl)hex-1-yne, which gave a mixture of isomeric vinylsilanes indicating that the steric effect of the silyl group plays a role, and extended reaction times gave reduced stereoselectivity. The resulting arylated vinylsilanes could be converted into their corresponding iodide or bromide. In the case of the iodide this was performed in a two-step, one-pot reaction sequence, whereas the bromide required two independent steps. In a further extrapolation of the chemistry the regio- and stereoselective synthesis of ( *Z* )-α-(4-tolyl)-β-(4-methoxyphenyl)styrene ( **102** ) was accomplished in three steps from 1-phenyl-2-(trimethylsilyl)acetylene. The *E* -isomer was prepared starting from 1-(4-tolyl)-2-(trimethylsilyl)acetylene (Scheme 80 ). The reaction was also possible with the addition of a vinylboronic acid giving a dienylsilane. 100


**Scheme 80** Addition of boronic acids to alkynylsilanes


The Oshima group reported the *syn* -hydrophosphination of terminal and internal alkynes. With arylacetylenes the regioselectivity was approximately 9:1 and with (triethylsilyl)acetylene, the sole silicon example, it was 94:6, slightly less than that with alkylacetylene substrates, which showed a 100:0 regioselectivity all placing the phosphine on the terminal position. The products were isolated as their phosphine sulfides (Scheme 81 ). 101


**Scheme 81** Hydrophosphination of (triethylsilyl)acetylene



**Scheme 82** Hydroalumination of 1-silylalk-1-ynes and asymmetric vinylation­ of enones


A chiral NHC catalyst was employed in the enantioselective conjugate addition of 1-(trimethylsilyl)alk-1-ynes to 3-substituted cyclopentenones and 3-substituted cyclohexenones. Thus, the 1-(trimethylsilyl)alk-1-yne was reacted with diisobutylaluminum hydride to form the 1-(trimethylsilyl)vinylaluminum reagent, which was then reacted with the enone, catalyzed by the chiral NHC complex **103** . In reactions with the cyclopentenones, up to 10% of addition of the isobutyl group from aluminum was observed; this increased to up to 33% for cyclohexenones. The er values were excellent, ranging from 92.5:7.5 to 98.5:1.5. Of considerable importance, the resulting vinylsilanes were further reacted. Oxidation with *m* -chloroperbenzoic acid gave the ketone. NCI converted it into the vinyl iodide and protiodesilylation to the parent alkene. This chemistry was applied to a short synthesis of riccardiphenol B ( **104** ) (Scheme 82 ). 102

The reaction of indoles with 1-(halophenyl)-2-(trimethylsilyl)acetylenes under Cu(I) catalysis gave addition of the indole to the C≡C bond and, under the basic conditions, protiodesilylation to form the corresponding alkene as a mixture of stereoisomers. Very little amination of the aryl halogen bond occurred. In fact, a control experiment wherein indole was reacted with a mixture of 1-(4-bromophenyl)-2-(trimethylsilyl)acetylene and 4-iodoanisole a 50% yield of addition to the C≡C bond and only 6% reaction of the iodophenyl­ bond was observed (Scheme 83 ). 103


**Scheme 83** Hydroamination of 1-(halophenyl)-2-(trimethylsilyl)acetylenes with indoles


The hydrosilylation of various propynoate esters was carried out and served to prepare α-silyl-α,β-unsaturated esters in good yields. When this reaction was performed with 3-(trimethylsilyl)propynoate esters, the product formed was the ( *E* )-2,3-bis(silyl)propenoate. Other similar systems such as the ynone **105** and sulfone **106** gave good yields of addition products (Scheme 84 ). 104


**Scheme 84** Hydrosilylation of functionalized silylacetylenes


1,4-Bis(trimethylsilyl)buta-1,3-diyne underwent carbomagnesiation of one of the C≡C bonds with arylmagnesium bromide reagents. The resulting vinylmagnesium bromide intermediate could be further reacted, including cross-coupling to form various substituted silylated enynes. 1-Phenyl-4-(trimethylsilyl)buta-1,3-diyne underwent carbomagnesiation at the phenyl-substituted C≡C bond (Scheme 85 ). 105


**Scheme 85** Addition of Grignard reagents to 1,4-bis(trimethylsilyl)­-buta-1,3-diyne


Kimura and co-workers reported on the Ni-catalyzed, four-component coupling of internal alkynes, buta-1,3-diene, dimethylzinc, and carbon dioxide. The reactions of 1-substituted 2-(trimethylsilyl)acetylenes gave lower yields and poorer regioselectivity than those of alkyl- or aryl-substituted alkynes (Scheme 86 ). 106


**Scheme 86** Four-component coupling involving 1-substituted 2-(trimethylsilyl)acetylenes


The three-component coupling of acetylenes, vinyloxiranes, and dimethylzinc was reported to give alka-2,5-dien-1-ols. Bis(trimethylsilyl)acetylene and (trimethylsilyl)acetylene gave lower yields than 1-(trimethylsilyl)prop-1-yne and alkyl- or arylalkynes. In a similar manner vinylcyclopropanes gave 1-silyl-1,4-dienes (Scheme 87 ). 107


**Scheme 87** Alkylative three-component coupling of silylacetylenes with vinyloxiranes and a vinylcyclopropane


The reaction of silylacetylene **107** by Ru-catalyzed addition of acetic acid gave a mixture of the desired enol acetate **108** along with **109** ; a longer reaction time gave **109** in good yield. Although **108** was the initial desired intermediate it was **109** that was in fact carried forward in a synthesis of clavosolide A (Scheme 88 ). 108


**Scheme 88** Hydroacetation of a silylacetylene


An iron-catalyzed imine-directed 2-vinylation of indole with internal alkynes produced the 2-vinylated derivative in good yield and regioselectivity. Terminal acetylenes did not react under the conditions employed. This deficiency was circumvented by the use of a (trimethylsilyl)acetylene, which reacted with high regioselectivity forming the C2–C _vinyl_ bond β to the TMS group. These conditions also proved useful for the formation of C2–Csp ^3^ bonds when the reaction was carried out with alkenes (Scheme 89 ); again the reaction did not occur with terminal alkenes. 109


**Scheme 89** Coupling of a (trimethylsilyl)acetylene with an α,β-unsaturated imine


The addition of DIBAL-H to 1-(trimethylsilyl)prop-1-yne followed by conversion into the lithium aluminate and reaction with formaldehyde resulted in vinylsilane **110** . This was in turn used to generated vinylsilane **111** and, from that, vinyl iodide **112** , which was then converted in two steps into norfluorocurarine ( **113** ) (Scheme 90 ). 110


**Scheme 90** DIBAL-H addition to 1-(trimethylsilyl)prop-1-yne


## 13 Reactions at the C–Si Bond

A study on the iododesilylation of a series of vinylsilanes wherein the silyl group included TIPS, TBS, and TBDPS was carried out. 111 This was the first report of the iododesilylation of a vinylsilane with sterically hindered silyl moieties. Interestingly, it was found that the rate of the reaction with TIPS or TBS groups was about the same, but that of TIPS was faster than that of TBDPS. Four different sources of I ^+^ , *N* -iodosuccinimide­ (NIS), *N* -iodosaccharin (NISac), 1,3-diodo-5,5-dimethylhydantoin (DIH), and bis(pyridine)iodonium tetrafluoroborate (Ipy _2_ BF _4_ ) were investigated with comparable results for each. The success of the reaction depended on the solvent system with 1,1,1,3,3,3-hexafluoropropan-2-ol (HFIP) showing good results. The reaction was tolerant of epoxides, alkenes, esters, TIPS ethers, and a TIPS acetylene (12 examples, 86–96% yield) (Scheme 91 ). 111


**Scheme 91** Iodination of vinylsilanes, readily available from silylacetyl­enes


1,4-Bis(trimethylsilyl)buta-1,3-diyne was treated with MeLi·LiBr to prepare the monolithiated diyne, which was reacted in a Sonogashira cross-coupling with 2-iodoaniline. The coupling product was reacted with trichloroacetyl isocyanate and this converted into desilylated urea **115** in a single step. The resulting diyne was subjected to a double cyclization to give the pyrimido[1,6- *a* ]indol-1(2 *H* )-one **116** (Scheme 92 ). 112


**Scheme 92** Lithiation of 1,4-bis(trimethylsilyl)buta-1,3-diyne



**Scheme 93** Ethynylation of α,β-unsaturated esters with (trimethylsilyl)acetylenes


Pan and co-workers reported the conjugate addition of alkynyl groups to acrylate derivatives via the reaction of a (trimethylsilyl)acetylene derivative under InCl _3_ catalysis. Silyl­ moieties other than that of the TMS group were not investigated. The reaction worked best for 1-phenyl-2-(trimethylsilyl)acetylene wherein the phenyl group is a strongly electron-donating aryl group. Thus, 4-CN-, 4-CO _2_ Me-, and 4-CF _3_ -substituted 1-phenyl-2-(trimethylsilyl)acetylenes failed to react. A direct comparison of 1-butyl- and 1-phenyl-2-(trimethylsilyl)acetylene with hex-1-yne and phenylacetylene, that is, the H-terminated acetylenes, showed that TMS-terminated acetylenes gave better yields. Chlorobenzene was found to be the best solvent and Et _3_ N the best base. 1,4-Bis[(trimethylsilyl)ethynyl]benzene ( **117** ) reacted with ethyl acrylate to give the mono- or disubstituted γ,δ-ethynyl esters. The reaction was also occurred with methyl vinyl ketone as the acceptor (Scheme 93 ). 113

This protocol compares well with the conjugate addition of terminal alkynes to acrylates catalyzed by Ru _3_ (CO) _12_ /bis(triphenylphosphine)iminium chloride and with Pd(OAc) _2_ . 114 115

## 14 Miscellaneous Reactions

β-Amino enone **118** was converted in a two-step, single-pot sequence into enol ether **119** via reaction with 3-(trimethylsilyl)propargyllithium in 51% overall yield; using propargylmagnesium bromide gave the corresponding H-terminated product in 40% yield. Enol ether **119** was utilized in a synthesis of 7-hydroxycopodine (Scheme 94 ). 116


**Scheme 94** Reaction of 3-(trimethylsilyl)propargyllithium with an iminium salt



**Scheme 95** Rearrangement of 1-(silylethynyl)cyclopropan-1-ols


1-[(Trialkylsilyl)ethynyl]cyclopropan-1-ols were ring expanded to 2-alkylidenecyclobutanones in a reaction catalyzed by the Ru catalyst **120** . Interestingly, the favored stereoisomer­ was the *Z* -isomer. Similar results were obtained with electron-deficient alkynyl cyclopropanols. On the other hand, under the same conditions 1-alk-1-ynyl­cyclopropan-1-ols underwent ring expansion to cyclopentenones. Stabilization of a β-carbocation in the silyl-substituted examples and a favored Michael addition in the electron-deficient examples helps to explain the formation of the four-membered ring (Scheme 95 ). 117

3-(Trimethylsilyl)propynal was nicely used in a convenient synthesis of ethynyl-β-lactone **121** ; propynal did not undergo a corresponding reaction to give **122** . The silylated enantiomerically enriched β-lactone **121** was utilized in synthetic approaches to leustroducsin B and the protiodesilylated ethynyl lactone **122** was converted to derivatives of similar structure to the natural products (–)-muricaticin, (–)-japonilure, and (+)-eldanolide. 118 119 120


**Scheme 96** Synthesis of silylethynyl-β-lactone


Corey and Kirst were the first to report the synthesis and utility of 3-(trimethylsilyl)propargyllithium ( **123** ). The direct lithiation of 1-(trimethylsilyl)prop-1-yne occurred using BuLi/TMEDA in 15 minutes. The reagent **123** reacted with primary alkyl halides in diethyl ether to form the desired alkynes with only small amounts of the isomeric allene, a common side product found with propargylmagnesium chloride reagent. 121

Corey and Rucker then utilized 1-(triisopropylsilyl)prop-1-yne ( **124** ), which was readily lithiated to give the more sterically encumbered 3-(triisopropylsilyl)propargyllithium ( **125** ). Lithium reagent **125** was reacted with cyclohexenones in a 1,2- and 1,4-manner. In addition it was converted into the 1,3-bis(triisopropylsilyl)prop-1-yne ( **126** ) in quantitative yield on treatment with triisopropylsilyl triflate. Reaction of **125** with cyclohexenone gave 1,4-addition in THF/HMPA and 1,2-addition in THF. Bis-TIPS reagent **126** reacted with BuLi/THF to give lithiated **126** , which reacted with aldehydes in a Peterson reaction to form an enynes (Scheme 97 ). 4


**Scheme 97** Formation and reactions of silylpropargyllithium reagents


3-(Trimethylsilyl)propargyllithium ( **123** ) was used to introduce the propargyl group into epoxygeranyl chloride in 85% yield over three steps from geraniol. The TMS group was removed with TBAF and the resulting enyne was used in a synthesis of the triterpene limonin (Scheme 97 ). 122

3-(Trimethylsilyl)propargyllithium ( **123** ) reacted with lactone **127** and this was followed by mesylation/elimination to give enynes **128** and **129** in good yield. The TMS group was removed with AgNO _3_ /aq EtOH en route to stereoisomers of bis(acetylenic) enol ether spiroacetals of artemisia and chrysanthemum (Scheme 97 ). 123

Fu and Smith demonstrated the enantioselective Ni-catalyzed, Negishi cross-coupling arylation of racemic 3-(trimethylsilyl)propargyl bromides; the yields and the ee values were excellent. The protocol was applied to the synthesis of **131** , a precursor to pyrimidine **132** , an inhibitor of dihydrofolate reductase (Scheme 98 ). 124


**Scheme 98** Asymmetric arylation of 3-(trimethylsilyl)propargyl bromides

